# Flexural and Shear Tests on Reinforced Concrete Bridge Deck Slab Segments with a Textile-Reinforced Concrete Strengthening Layer

**DOI:** 10.3390/ma13184210

**Published:** 2020-09-22

**Authors:** Viviane Adam, Jan Bielak, Christian Dommes, Norbert Will, Josef Hegger

**Affiliations:** Institute of Structural Concrete, RWTH Aachen University, 52074 Aachen, Germany; jbielak@imb.rwth-aachen.de (J.B.); cdommes@imb.rwth-aachen.de (C.D.); nwill@imb.rwth-aachen.de (N.W.); jhegger@imb.rwth-aachen.de (J.H.)

**Keywords:** reinforced concrete, bridge deck slabs, strengthening, textile-reinforced concrete, carbon concrete, static loads, fatigue loads, experimental investigations

## Abstract

Many older bridges feature capacity deficiencies. This is mainly due to changes in code provisions which came along with stricter design rules and increasing traffic, leading to higher loads on the structure. To address capacity deficiencies of bridges, refined structural analyses with more detailed design approaches can be applied. If bridge assessment does not provide sufficient capacity, strengthening can be a pertinent solution to extend the bridge’s service lifetime. For numerous cases, applying an extra layer of textile-reinforced concrete (TRC) can be a convenient method to achieve the required resistance. Here, carbon fibre-reinforced polymer reinforcement together with a high-performance mortar was used within the scope of developing a strengthening layer for bridge deck slabs, called SMART-DECK. Due to the high tensile strength of the carbon and its resistance to corrosion, a thin layer with high strength and low additional dead load can be realised. While the strengthening effect of TRC for slabs under flexural loading has already been investigated several times, the presented test programme also covered increase in shear capacity, which is the other crucial failure mode to be considered in design. A total of 14 large-scale tests on TRC-strengthened slab segments were tested under static and cyclic loading. The experimental study revealed high increases in capacity for both bending and shear failure.

## 1. Introduction

The age distribution of the existing bridges in combination with several normative changes and significant economic and demographic changes in many industrial countries lead to many structures showing damages. Or else, there are computational deficits due to increased traffic or stricter verification [1,2,3,4,5,6,7]. In order to extend the remaining service life of the currently deficient structures, refined design concepts can provide a remedy that allows higher computational load-bearing capacities. In many countries, strategies have been developed for bridge assessment including monitoring, maintenance and design evaluation [8,9,10,11,12,13]. The aim is to extend the planning horizon in order to postpone the construction of a new replacement bridge for at least a part of the affected bridges. If some of the required verifications cannot be met even after a more detailed structural analysis during bridge assessment, strengthening measures can provide pertinent solutions. For reinforced or prestressed concrete bridges, which represent the majority of the German bridge population [3,14], established strengthening methods are available, such as additional external prestressing [15], insertion of shear connectors, additional concrete in the compression zone or external application of CFRP sheets or lamella [16,17,18]. Innovative materials, such as UHPC [19,20,21,22] or textile-reinforced concrete (TRC) [23,24,25] enable considerable material savings through increased performance. This results in a more resource-efficient use of materials with a reduced additional dead weight and an extended service life. In consequence, the substructures and foundation of the strengthened bridge are exposed to less extra loading while CO_2_ emissions from initial construction are spread over a longer period of use. Thus, the ecological footprint of concrete structures might be improved.

The findings presented in the following were part of a project aiming at developing a thin TRC layer which is intended to be added on bridge deck slabs between the RC structure and the road surface. It is called SMART-DECK and offers a three-fold functionality comprising monitoring, corrosion protection and strengthening of the transverse system of concrete T-beam or hollow core bridges. Strengthening is subject to the present paper. In terms of the gain in capacity due to the additional TRC layer, not only regular flexural and but also shear tests were conducted. The results of the experimental investigations are presented later. Prior to the presentation of the conducted investigations, the concept of the strengthening layer is described in Section 2. At the beginning, basic information on TRC is provided to give an understanding of the choice of material and the layout of the TRC layer.

The aim of the study is outlined in detail in Section 2.2, describing the specifics of SMART-DECK and outlining its distinction towards other studies addressing strengthening using TRC. The experimental programme is introduced in Section 3, beginning with small-scale tests. In Section 4, large-scale tests on slab segments under static and cyclic loading are presented. The paper concludes in Section 5 with a brief summary and prospects regarding potential future investigations.

## 2. Concept of the Strengthening Layer

### 2.1. Textile-Reinforced Concrete

Construction with reinforced concrete is rather economical and characterised by its versatile shaping. The two main elements—concrete and steel reinforcement—are combined to exploit their full potential when interacting with each other. One main disadvantage is the predisposition of the steel reinforcement for corrosion. If the concrete surface is damaged or chlorides enter the structure, the reinforcement can be damaged despite sufficient concrete cover. For this reason, research is concerned with the development of corrosion-resistant reinforcement elements. In the last two decades, research on corrosion-resistant textile reinforcements has been intensified. Early applications from Japan on non-metallic carbon mesh reinforcement [26] inspired research in the Collaborative Research Centre (*Sonderforschungsbereich*, *SFB*) 528 at TU Dresden, Germany, on strengthening of existing structures [27,28,29,30], while fundamental research on new construction elements utilising this then new technology was conducted in SFB 532 at RWTH Aachen University, Germany, [31,32,33,34,35].

**Figure 1:**
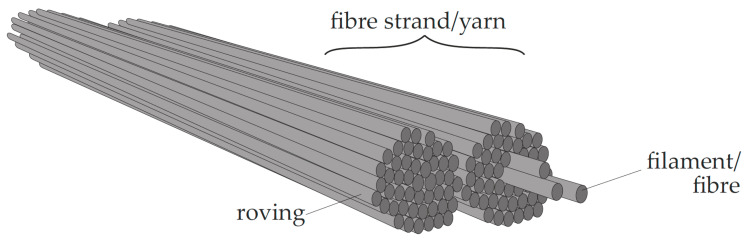
Principal sketch of a larger number of single filaments assembled to a roving and then to a yarn.

The reinforcement in TRC is comprised of continuous fibres, which are called filaments (Figure 1). These are characterised by their good mechanical properties such as high tensile strength and, depending on the base material, medium to high modulus of elasticity. Depending on the desired fineness and the application, up to thousands of filaments are assembled to form a roving. The most common production method for non-crimp reinforcements is warp-knitting, where one or multiple rovings are arranged equidistantly and fixed in their position by additional knitting yarns, forming two-dimensional (2D) mesh-like reinforcement textiles. In biaxial textiles, warp rovings run in the longitudinal direction of the mesh, while weft rovings are arranged perpendicular to it.

Alkali-resistant (AR) glass fibres and carbon fibres are preferably used for TRC. Both materials have good mechanical properties, but carbon fibres are more durable than AR glass fibres [36]. Carbon fibres are characterised by their low density, a very high tensile strength and a high modulus of elasticity. They are manufactured in a multi-stage thermal process which determines the strength and stiffness of the fibres. Polyacrylonitrile (PAN) is usually used as the starting material for carbon fibres [37].

Bond of non-impregnated textiles is achieved by adhesion and friction rather than mechanical interlock, which is the typical mechanism for conventional deformed steel rebar. Due to the circular shape of the filaments and their small diameter, small voids exist in the roving. Because of their small size, cementitious binder cannot penetrate the roving and only encloses the edge filaments. The core filaments can only be activated for load transfer by friction on the edge filaments. Due to the smooth surface of the fibres, this bonding mechanism is ineffective and results in a telescopic failure with a stress gradient over the cross-section. An impregnation with low-viscosity organic or mineral binders fills the voids and allows for simultaneous activation of the core and sleeve material [34,38]. The most common impregnation materials are epoxy and styrene-butadiene. Also, new concepts with cementitious impregnation exist [39]. While meshes with styrene-butadiene impregnation maintain a certain flexibility but feature a rather soft bond to the concrete or mortar matrix, an epoxy resin provides a relatively rigid material behaviour which forms a strong bond to the cementitious matrix. At the same time, the latter allows for a much more straight-forward production since the reinforcement can be assembled in a formwork similar to reinforcing steel and the concrete can be regularly cast. It only needs to be ascertained that buoyancy of the textiles during compaction is prevented. Also, textile reinforcement must not be walked on due to its sensitivity to lateral compression. To a certain extent, this makes CFRP textiles with epoxy impregnated carbon rovings comparable to carbon FRP reinforcement. After concreting, it can be summarised under the term carbon concrete. The high potential of carbon concrete as an effective construction material has already been given prove of in numerous research projects and applications [40,41,42,43,44,45,46,47].

### 2.2. Characteristics of SMART-DECK

#### 2.2.1. Intended Functions and Main Questions

**Figure 2:**
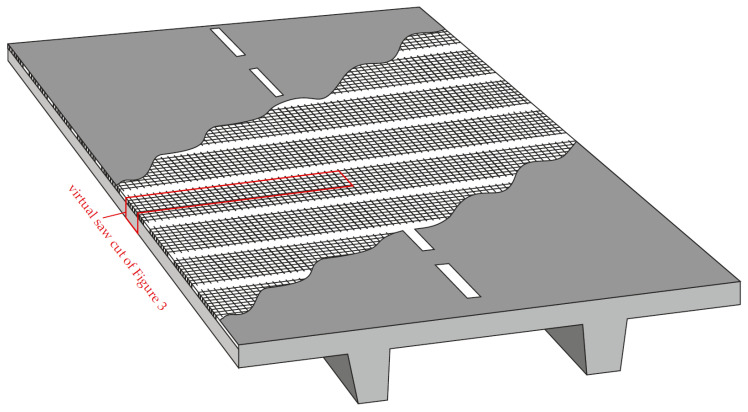
Position of the additional textiles on an exemplary bridge deck slab.

The system SMART-DECK presented here was developed in a research project in cooperation with a total of seven project partners from research and building practice as well as administration and material production. It is added to the top of the bridge between the concrete slab and the road surface (Figure 2) and enables bridge strengthening with TRC and offers two other functionalities which were investigated by the project partners: moisture monitoring and preventive cathodic corrosion protection (pCCP) [48,49]. The overall investigations not only foculised the target functionalities but also the feasibility and manufacturing of the system on a bridge [50]. The strengthening effect was investigated at the Institute of Structural Concrete of RWTH Aachen University (IMB) and it is the central content of this paper. In contrast to other investigations with regard to the strengthening potential of bridges with TRC, SMART-DECK not only addresses flexural but also shear capacity of the bridge’s transverse system. These represent the main failure cases in ultimate limit state design of the bridge deck slab. So far, the impact of shear strengthening with TRC has been investigated with respect to the longitudinal system of the bridge [25,51,52,53], i.e., by supplementing the cross-section of the stirrup-reinforced webs. Since shear behaviour of slabs without shear reinforcement differs from that of beams with shear reinforcement [54], insights gained from previous results cannot simply be transferred to the transverse system. Comparable slab strengthening systems [55,56,57] addressed the increase in flexural capacity. For bending, significantly more pronounced tensile stresses can be assumed in the textile reinforcement compared to shear being the critical load case. Since the very high tensile strength is the essential characteristic of the carbon reinforcement, the question remained to which extent a significant increase in capacity could be achieved by increasing the flexural reinforcement when premature shear failure occurs.

Another research question was the manufacturability of SMART-DECK. A common method for TRC production is shotcreting [52,58,59]. Since SMART-DECK is realised in a horizontal plane, shotcreting is not a suitable solution due to ingress of rebound. Therefore, using shotcrete would have conflicted with technical rules for German bridges [60] in this application and was therefore excluded. A production via laminating (layer by layer) is labour-intensive, so regular concreting was preferred from the beginning. Initially, it was not clear whether this method would meet the various technical demands resulting from the implementation of the instrumentation for monitoring and pCCP or a possible device for (partially) automated manufacturing. The manufacturing method was also linked to the development of suitable spacers and fasteners for the reinforcement which do not interfere with the electrical current.

#### 2.2.2. Layout

**Figure 3:**
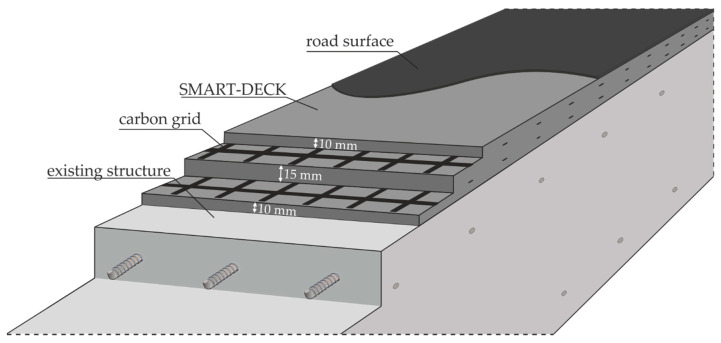
Virtual saw cut as schematic sketch of the strengthening layer on a RC slab.

SMART-DECK is a supplementary TRC layer, which is applied on top of the existing concrete and subsequently covered by the road surface (Figure 3). It is to be processed along the entire width of the bridge deck, but the textiles are to be laid in sections in longitudinal direction of the bridge at a defined distance from each other in order to obtain electrically separated fields. SMART-DECK consists of a high-performance mortar and a textile reinforcement made of carbon fibres impregnated with epoxy resin. The textiles are installed in two layers with a mutual centre distance of 15 mm and an edge distance to the concrete interface and to the upper side of 10 mm, resulting in a total layer depth of approximately 35 mm.

The bond between strengthening layer and existing concrete in this system is not achieved by mechanical connectors but relies on concrete-to-mortar adhesion. This greatly reduces application time and cost but requires proper surface preparation prior to casting as well as a close quality control on site. The basic material carbon of the TRC layer offers the electrical conductivity required for monitoring and pCCP on the one hand. On the other hand, in combination with epoxy resin impregnation, it has good bonding properties to the surrounding concrete and is very efficient due to its high axial tensile strength (5 to 6 times the value of reinforcing steel). The corrosion resistance of the material allows the execution of thin layers with small concrete covers. Especially in combination with a massive existing supporting structure, which provides high stiffness, these advantageous properties of TRC can be used optimally and high increases in load-bearing capacity can be achieved with a minimum use of material.

### 2.3. Previous Investigations

As stated before, the flexural strength of regular RC components with small longitudinal reinforcement ratios can be significantly increased by TRC strengthening in the flexural tension zone [57,61,62,63]. Investigations on the influence of web strengthening with TRC showed that shear capacity of reinforced concrete beams can also be significantly increased by TRC strengthening [23,24,52]. Here, in addition to the high tensile strength of the textile reinforcement, the reduction of the crack width plays an important role, since this can prevent an early confinement of the flexural compression zone and delay the propagation of the shear crack into the compression zone. Furthermore, it could be shown that web strengthening with TRC can also be a useful alternative to existing strengthening methods for cyclically stressed components [23,53,64].

Prior to the start of the project, tests were conducted to estimate whether promising degrees of strengthening for the main failure modes in transverse direction of the bridge can be achieved in the ultimate limit state [65]. It was shown that a supplementary TRC layer can increase shear capacity of the bridge deck. However, different materials and different boundary conditions were applied in those tests compared to the investigation presented in this paper. Therefore, these results only allowed for tendentious statements.

The strengthening effect of SMART-DECK itself was already experimentally examined during realisation of the project demonstrator. For this purpose, a slab of approximately 100 m^2^ was built which also met the demands resulting from the other two intended functionalities. It featured a height of *h* = 28 cm and a change of slope was produced to represent realistic conditions for fabrication with respect to an existing bridge slab. The additional TRC layer was applied to 80% of the area, whereby the implementation of the cross-sectional supplement itself and the manufacturing of the measuring device for the monitoring system were tested. Subsequently, the monitoring system (see above) and the achievable increases in capacity were investigated. The latter was done by extracting saw-cuts from the strengthened and non-strengthened areas of the demonstrator slab, which were then tested in a total of eight load tests at IMB until failure. Bending and shear were investigated in the same way as in the series of tests presented here: 24–56% shear strengthening and 90–174% flexural strengthening were achieved [64].

## 3. Experimental Programme and Results of Small-Scale Tests

### 3.1. General

**Figure 4:**
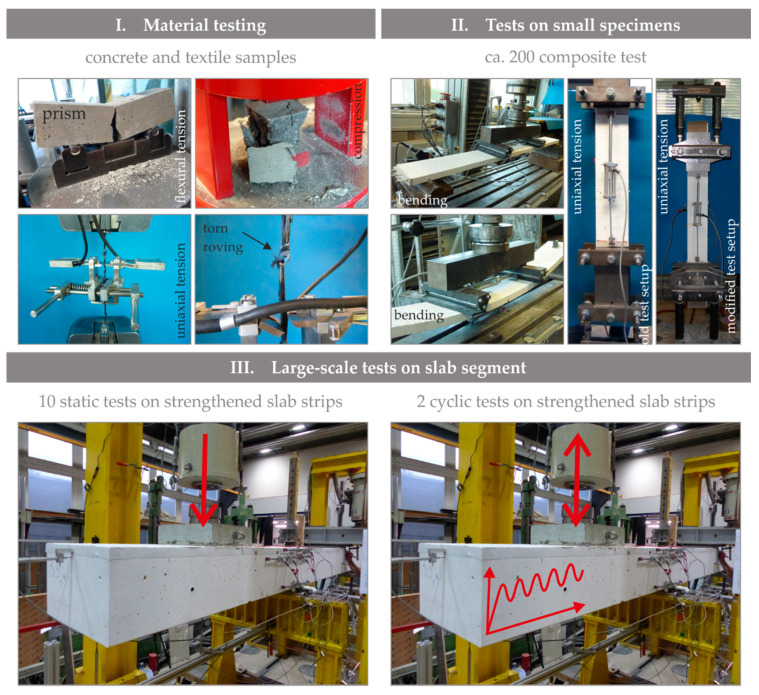
Overview of the experimental programme (photographs by IMB, RWTH Aachen University).

Material and small-scale tests were carried out to characterise the materials intended for SMART-DECK and their interaction with each other. The goal was to select a suitable combination of mortar and textiles for strengthening (I. and II., according to Figure 4). The small-scale tests were divided into three groups: uniaxial yarn tensile tests as well as flexural and uniaxial tensile tests on composite strips. In the uniaxial yarn tensile tests, individual fibre strands extracted from the mesh were investigated, while in the bending and other uniaxial tensile tests, composite components with textile cut-outs embedded in concrete were tested. Also, mortar testing was performed on each batch that was used for SMART-DECK on prisms *W* × *H* × *L* = 40 × 40 × 160 (mm) in accordance with Reference [66].

**Figure 5:**
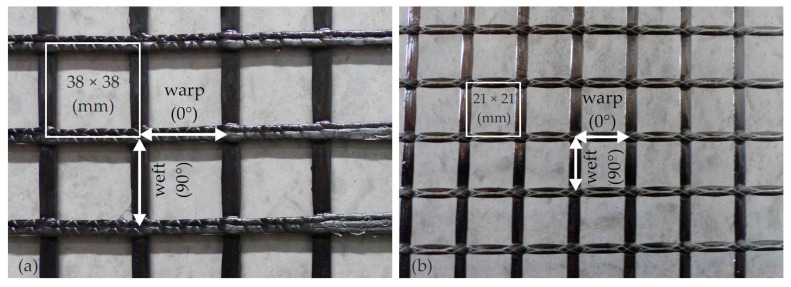
Textile grids made of carbon with epoxy impregnation with a mesh size of 38 mm (**a**) and 21 mm ((**b**), photographs by IMB, RWTH Aachen University).

The quadratic textile reinforcement meshes were provided by the project partner solidian. The mesh size (centre-to-centre distance of the fibre strands) was either 21 or 38 mm. Depending on the knitting method, different bond properties can occur which not only differ among the various grids but also depend on the direction [38] since weft and warp vary in cross-sectional shape and surface characteristics (Figure 5).

With the findings from the small-body tests, in which various parameter combinations were investigated, the materials for the final SMART-DECK system were pre-selected. This optimised combination was then used as a strengthening layer in the large-scale tests. Small-body tests are particularly suitable for this purpose, as multiple repetitions with the same combination of properties and a large number of different specimens can be tested in a comparatively short time. The tests were labelled X-YZ based on the type of experiment and the materials used as follows:

- X: test type (B: Bending, T: Tensile)
- Y: applied mortar (consecutive number from the material notation)
- Z: applied textile (consecutive number from the material notation)

For some tests, another abbreviation was added to the end in order to mark a more specific property. Table 1 shows the materials used in the course of the investigations on the strengthening effect of SMART-DECK. The notation A-B-C of the materials results from the project consortium’s definitions, where A is the material (M: mortar, T: textile), B is a consecutive number for each material and C is a specification (maximum aggregate size, in mm, for mortar and mesh size, in mm, for textiles).

Due to the production process, the weft direction of the textiles is limited to the width of the textile machines. For this reason, the weft direction is positioned in longitudinal direction and the warp direction in transverse direction of the bridge (main load transfer direction of the roadway slab) since separated segments of textile are required in longitudinal direction by means of electrically insulated areas (Figure 2). While the flexural and tensile tests on the composite material were therefore tested in warp direction, the yarn tensile tests were carried out for both fibre strand directions in order to obtain comparative values. Figure 4, Step I, shows the test setups and measuring technology used for the tensile tests on individual fibre strands and an example after failure.

### 3.2. Uniaxial Tensile Tests on Individual Fibre Strands

For test preparation, the fibre strands were extracted from the grid approximately at the centre between two strands (10–15 mm from the stand axis). The textiles have comparatively large yarn cross-sections. At the same time, carbon filaments have high strengths, so high failure loads had to be assumed, which required high lateral compression in the clamps at the ends of the yarns. To prevent them from rupturing in the clamps, the ends were glued into aluminium foil with the aid of epoxy resin mortar. These straps were fixed between the jaws of the clamps after curing.

**Figure 6:**
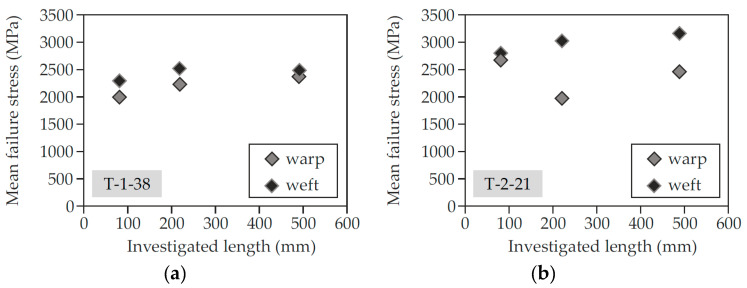
Results of tensile tests on yarns for T-1-38 (**a**) and T-2-21 (**b**).

Only results from tests were considered where the yarn actually ruptured within the free length. Loading was applied displacement-controlled at 1 mm/min. Meanwhile, the applied tensile force and the strain were recorded. Figure 6 shows the results for tests on T-1-38 (left) and T-2-21 (right) as mean value of 5–10 samples.

The results differ with regard to the test length (free length between the clamps). Since the probability of imperfections increases with increasing length of investigation, its influence should be checked. For T-1-38, no negative influence due to increasing test length is evident. Only in weft direction, slightly smaller stresses do occur at the largest test length compared to the medium test length. Instead, the tests on the weft yarns of T-2-21 show increasing ultimate stresses with longer test length. Only in warp direction of T-2-21, a decreasing trend is visible, whereby the tests with medium lengths showed comparatively low tensile strengths. Overall, the ultimate stresses of T-1-38 are somewhat lower than those of T-2-21 and the fibre strands in the weft direction have higher tensile strengths than those in the warp direction, which is due to the negative influence of the knitting thread and is commonly known [67,68].

### 3.3. Small-Scale Tests on the Composite

As mentioned before, the composite system was tested in small-body tests under bending and uniaxial tension (Figure 4, step II) to determine the essential properties of the strengthening layer under tension, such as cracking behaviour and stress–strain relationship. The pure tensile stress approximately corresponds to the eventual stress of the TRC layer on the bridge. As considerably more tests and thus more parameter combinations could be tested in a shorter time with bending tests, this method was preferred at the beginning of the project. The initial setup for uniaxial tensile tests ((Figure 4, step II), left test setup for tensile tests) required manual fixing of the specimens between the clamping jaws using several threaded rods, all of which had to be uniformly tightened. For the first materials tested within the scope of the project (M1, M2, T1 and T2 according to Table 2), both methods were therefore used. The dimensions of the specimens were determined according to the geometric properties of the textiles and the layered structure of SMART-DECK. Therefore, one layer of textile was bi-symmetrically positioned. The width of the composite strip was the multiple of the textile’s mesh width and the depth was 20 mm to represent a segment taken from a wide strengthening layer.

The textiles were placed in concrete over a total length of 1000 mm. For the flexural test, the TRC strips were supported over a span of 900 mm, with support overhangs of 50 mm at both ends. The test set-up was a 4-point bending test with centre distances of 300 mm in relation to the position of the supports and load application points. Large deflections occurred (100 to 130 mm in the middle of the span at ultimate load), which correspond to five to six times the depth of the test specimen. Failure always occurred via a propagating crack by spalling of the concrete compression zone without rupture of the textile. As the reinforcement did not fail and due to second-order effects (large deflection of the specimens in relation to the horizontal axis of support), the results of the bending tests were not taken into account in the evaluations and only uniaxial tensile tests were used in the further investigations of the composite load-bearing behaviour.

Further development of the uniaxial tensile test setup [69] minimised execution time. As shown in Figure 4, step II, on the right, the clamping jaws were replaced by a hydraulic device that simplified the installation of the specimens considerably. This setup was used from series 2 onwards. It can be concluded that flexural tests are unfit to determine the mechanical properties of composite specimens that are very thin and simultaneously feature such high flexural slenderness, *l*/*d* (here: *l*/*d =* 90). In this case, the tensile test setup does provide convenient results.

**Figure 7:**
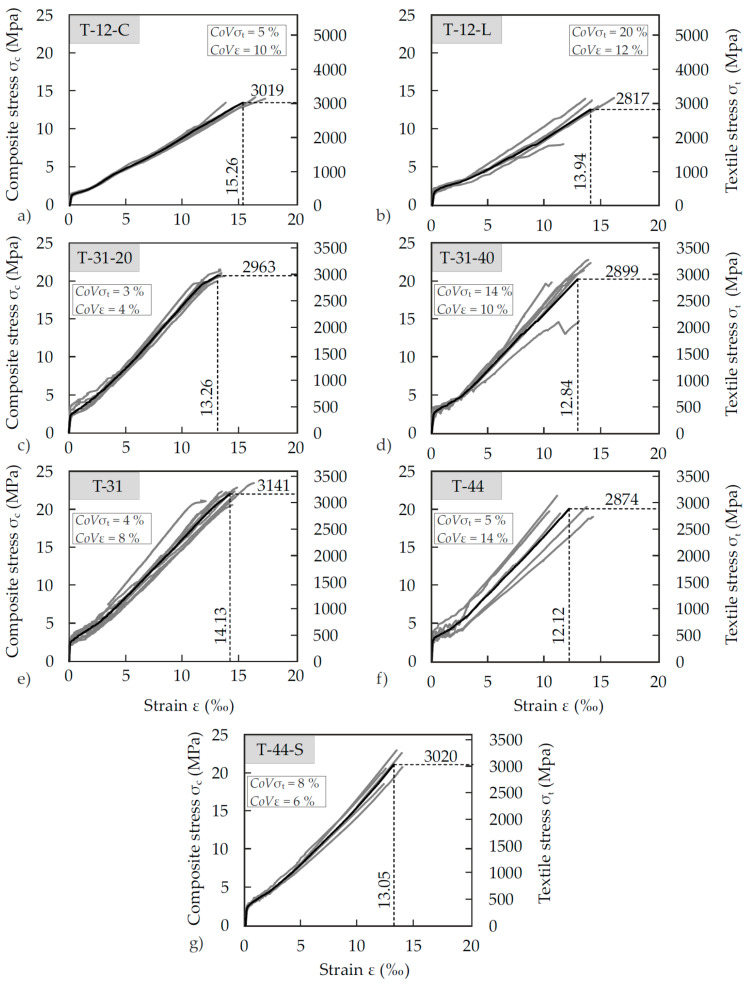
Stress–strain relations of seven exemplary groups of uniaxial tensile tests on composite strips with seven different material combinations (**a**–**g**) and indication of the scatter of ultimate stresses, σ, and strains, ε, by means of the coefficient of variation (CoV).

A total of five test series was investigated with their main parameters depending on the progress and open questions regarding the efforts of the project partners to develop advanced materials that meet all, and sometimes contradictory demands resulting from the different targeted functions of SMART-DECK (monitoring, pCCP and strengthening). While all tensile tests aimed at receiving essential insights on mortar to reinforcement combination, each series also aimed at defining another parameter, like, for instance, the following:

- Series 1: manufacturing method → half the specimens were regularly cast (indication C in Figure 7a) with the mortar being fluid while the other half was manually laminated (indication L in Figure 7b) using a stiffer version of the mortar.
- Series 2: number of textile layers → two layers were tested as in the actual strengthening layer (indication 40 mm depth) or one layer as in the description above (indication 20).
- Series 3: water-to-cement ratio of the mortar.

Figure 7 shows the results of some of the uniaxial tensile tests on composite strips by means of their stress–strain relations. While the tensile force is related to both the composite and the textile area (left and right y-axis, respectively), the grey curves show the individual result of one test and the black line represents the mean curve calculated from the total of the individual results correspondent to an established procure [69]. Thus, it was determined by averaging stress values of all test curves over predefined equal strain intervals until the first curves end due to failure of the corresponding specimen. To define the mean increase in length at failure, an additional data point was defined which corresponds to the average strength of the individual specimens.

**Figure 8:**
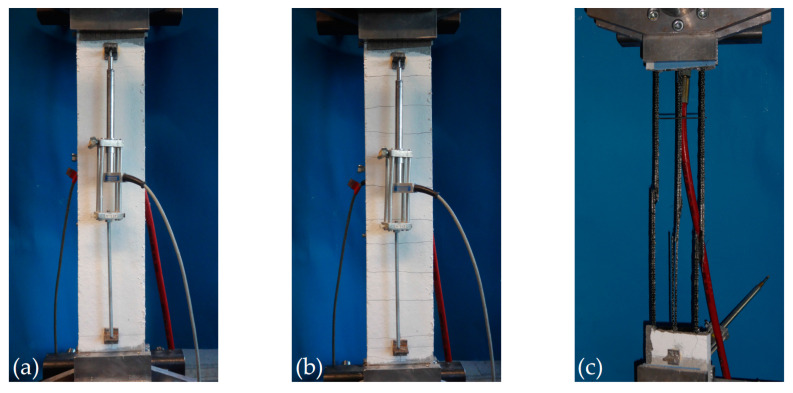
Stages of a tensile test: installed test specimen with slight cracking (**a**), specimen featuring complete crack formation (**b**) and specimen after failure (**c**), photographs by IMB, RWTH Aachen University).

Those and the other tensile tests that are not shown in detail here, were conducted to characterise the properties in the warp direction, which is decisive for the strengthening effect in the present case. The specimen length was 1000 mm, and the anchor length at each end was 250 mm. Figure 8 shows a uniaxial tensile test until the specimen failed. The cumulative crack openings were measured over a length of 450 mm using displacement transducers attached to the front and back to derive the mean strain of the reinforcement. This is possible because of the large number of cracks in the measurement area (significantly more than five cracks, as indicated in [69]). The tests were carried out displacement-controlled at 2 mm/min.

Due to the specified layered structure of SMART-DECK with a concrete cover of 10 mm, the concrete cover of the tensile strips was also limited and not optimised regarding maximisation bond or maximum tensile stress development. Therefore, it was expected that a full exploitation of the material’s potential might not succeed, which was confirmed by the tests. After completion of the crack formation transverse to the load direction, longitudinal cracks often formed in the textile plane. Failure of the fibre strands occurred abruptly and was accompanied by concrete spalling. In some cases, there was no or only partial spalling. In these cases, however, the matrix clods were no longer bonded to the textile. The fibre strands in tensile direction either ruptured or remained partially intact. Since the specimens that were concreted by means of pouring and compacting (Figure 7a) featured higher ultimate loads and less scattering than comparable specimens that were laminated (Figure 7b), the former manufacturing method was used for the following tests. It was also the preferred procedure in terms of ease-of-use since it is easier and faster to apply to large surfaces like bridge decks. In terms of the specimens’ depth, no significant difference was observed (Figure 7c,d). Therefore, specimens that only had one layer of textile reinforcement allow for a representative investigation of the tensile resistance of SMART-DECK. Material combinations 31 and 44 (Figure 7e,f) were used as strengthening layers for the large-scale tests described in Section 4.

In the first two mortars tested, the fibre strands generally failed smoothly. In the more advanced mortars, they were mostly frayed. An improvement in the bond properties was achieved by further development of the textiles (T-4-38). The highest failure stresses, and at the same time the smallest dispersion, were obtained in tests with sanded textiles of T-4-38 in combination with M-4-04 (Figure 7g). However, since sanding results in considerably poorer electrical properties, it was not shortlisted for the project. T4 featured the overall best properties regarding all three target functions of SMART-DECK and was therefore chosen for the large-scale tests.

## 4. Large-Scale Tests

### 4.1. Design and Materials

For investigation of the strengthening effect of SMART-DECK, a total of 17 results from component tests are available, as the overview in Table 2 shows. Twelve of them were carried out on strengthened slab segments. From another project, a double reference test with a longitudinal reinforcement ratio of ρ_l_ = 1.0% can be referred to [70]. The aim was to investigate the influence of SMART-DECK on the component behaviour for the two decisive failure types, bending (M) and shear (V), as well as failure in the interface (I). In Table 2, a distinction is made between planned and occurred failure types. For mixed forms, the sequence corresponds to the sequence of failure modes in the test. For example, V + I means that primary failure was due to flexural shear and the interface failed secondarily.

**Figure 9:**
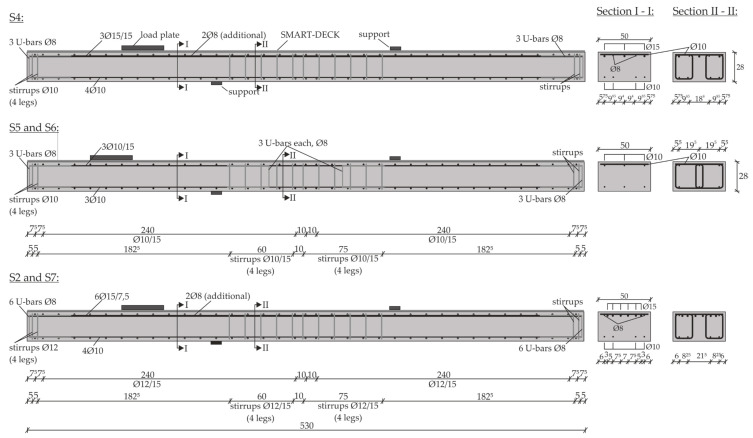
Outline of the reinforcement of the strengthened specimens S2, S4, S5, S6 and S7.

To control the bending moment, the load distance *a* = 0.7/1.0/1.3 m between load and support was modified and to additionally vary the exploitation of the flexural reinforcement, the longitudinal reinforcement ratio was set to either ρ_l,s_ = 1.0/0.5/0.2% in terms of the steel in the existing slab. For the high and medium longitudinal reinforcement ratio, threaded steel bars with a strength class of St900/1000 (Ø15) were used, and for the small longitudinal reinforcement ratio, a ribbed steel of B500 quality (Ø10) was used. The reinforcement layout of the reinforced concrete slab segments is shown in Figure 9. One half of specimen S8 was reinforced like S2 and S7, while the other half was equal to the reinforcement of S5 and S6, respectively. The non-strengthened reference slab segments featured a reinforcement that was identical to the corresponding specimens with TRC-strengthening.

No shear reinforcement was provided in the shear spans, as its installation in slabs is costly and therefore unusual in trunk road bridge slabs for practical reasons. A longitudinal reinforcement of Ø10/20 in transverse direction to the load transfer direction of the test bodies (longitudinal direction of the bridge) was provided in accordance with the normative minimum value for bridge slabs. The height of the RC base specimens was 28 cm, and the concrete cover was 20 mm all around. All slab segments were designed with a width of *b* = 50 cm, while the load plate’s widths were 40 mm to involve the entire cross-section in load transfer. No significant impact was expected by the additional TRC layer on stress redistribution in the slab originating from the concentrated load. In the near past, the influence of the slab width was extensively investigated for reinforced concrete slabs [71,72,73,74,75] and it is assumed that the findings are generally applicable to TRC strengthened slabs.

The reinforced concrete base bodies were concreted indoors with a ready-mixed concrete featuring a target strength corresponding to a C30/37 and a maximum aggregate size *d*_g_ = 16 mm. The specimens were compacted by means of an internal vibrator. Material samples (cylinder Ø = 150 mm/*h* = 300 mm and cubes with an edge length of 150 mm) were produced, which were stored next to the slab segments and tested at the time of the component test in order to be able to draw conclusions about the developed concrete strengths of the slab specimens (Table 2). Prior to the application of the strengthening layer, the upper surfaces of the reinforced concrete components were roughened by means of solid blasting. Using the sand surface method [76], a mean roughness of at least *R*_t_ = 1.0 mm was determined (three measurements per slab segment). SMART-DECK was manufactured by the project partner Eurovia under construction site conditions.

Specimens S4, S5, S6, S7 and S8 were produced together and strengthened some weeks later at once. They had the same material combination in the TRC layer. The specimen which was used for tests S2-1 and S2-2 was manufactured at an earlier stage with the material combination then available.

### 4.2. Test Setup

**Figure 10:**
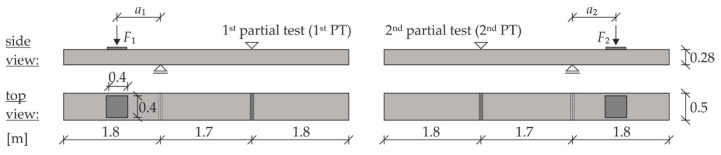
Specimen dimensions and test setup for the large-scale tests on slab segments (graphic by IMB, RWTH Aachen University).

Two separate tests were carried out on each specimen (Figure 10). The load was always applied to represent a load resulting from a truck driving on the outer lane and thus loading the cantilever of the bridge slab. The load distance, *a_i_* (load axis to axis of the support close to the load), was varied according to the specifications in Table 2. The support with a larger distance to the load was designed to take the lifting forces. The load was applied via a hydraulic cylinder and transferred to the specimen via a square load plate of 40 × 40 (cm) corresponding to the wheel contact area for trucks according to European standard [77].

All tests were statically loaded with stepwise increments until failure, except S8-1 and S8-2, which were loaded cyclically. The first four load stages were introduced load-controlled. From about half of the expected failure load, the load was applied deformation-controlled.

### 4.3. Results of Tests with Static Loading

#### 4.3.1. Cracking

In some tests, there were production-related imperfections in the interface between old and new concrete. Therefore, the TRC layer was partially detached from the existing concrete during the tests. However, these delaminations only occurred in the concrete interface and always originated from pre-existing imperfections. In case of intact interfaces, the load did not cause the joint to open, which could already be observed in previous tests [78].

**Figure 11:**
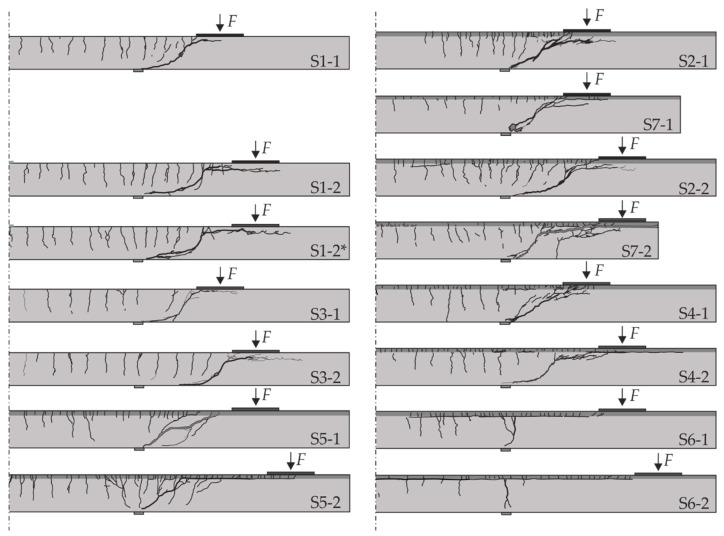
Crack patterns of static tests on specimens S1 to S7.

Figure 11 shows the crack pattern the specimens exhibited after failure. During loading, a finely distributed crack pattern developed in the TRC layer at the top of the specimens (tension zone). The significantly larger number of cracks with simultaneously reduced crack widths in the strengthened components in comparison to the non-strengthened reference tests is to be regarded positively with respect to the influence of SMART-DECK on the serviceability.

**Figure 12:**
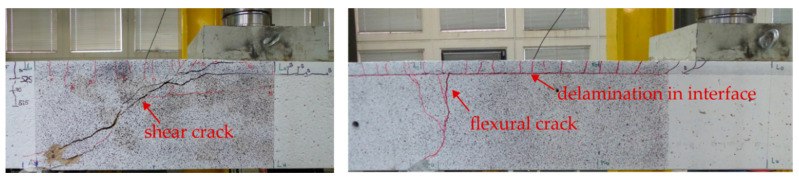
Essentially distinguished cracks related to failure (photos and graphic by IMB, RWTH Aachen University).

Based on the crack patterns, the tests can essentially be divided into three groups, differentiated by primary cracking that introduced failure, or could be observed at the time when ultimate load was reached (Figure 12). Flexural failure was characterised by a wide vertical accumulated crack in the area of the support (test S6-1 and S6-2 in Figure 11). Relatively few other cracks occurred in the base body. In case of shear failure, an inclined flexural shear crack emerged, which exhibited a large crack opening at failure. This crack pattern was observed among the majority of tests in Figure 11, including the reference specimens. If delamination in the concrete interface occurred prior to failure, the shear crack usually propagated along the flexural reinforcement of the RC structure and the propagation of the crack did not continue into the TRC layer. In case of an intact interface between the existing structure and SMART-DECK, the crack propagated into the interface or the strengthening layer. Then, considerably higher ultimate loads could be achieved than in the case of primary interface failure, which will be discussed in more detail later.

With a few exceptions, activation of the carbon concrete supplement could be achieved in the tests presented here, as shown in the previous section. However, the findings from manufacturing the strengthening layer for the specimens of this experimental programme provided crucial indications for the further development of the mortar and the production methods. In subsequent applications [50], unimpaired bonded interfaces were achieved so the complete functionality of the system could be realised.

#### 4.3.2. Load-Bearing Behaviour

**Figure 13:**
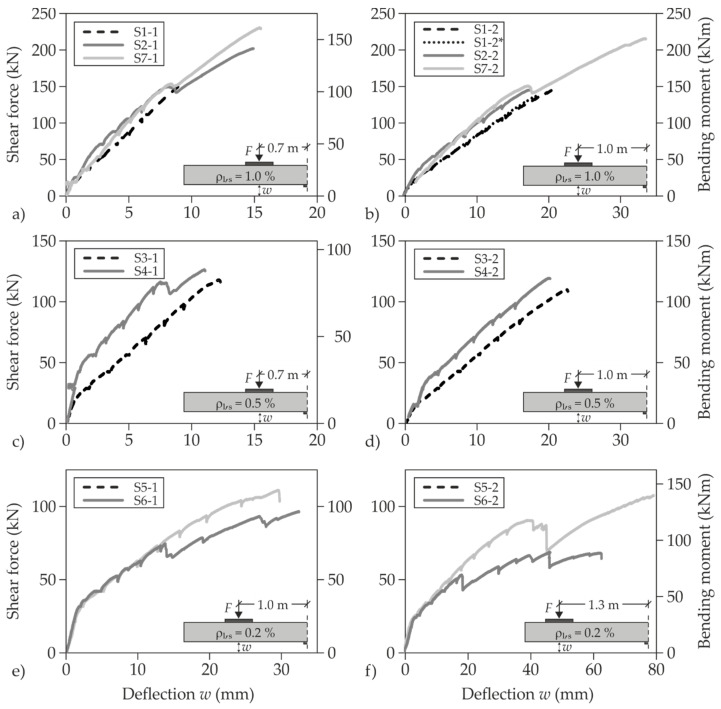
Load deflection curves of large-scale tests on slab segments with static loading (**a**–**f**) separated by combination of flexural reinforcement and load distance.

In addition to crack formation, SMART-DECK influences the load-deflection behaviour of the slab segments (Figure 13 for static tests). Here, a differentiation was made according to the load distance, *a_i_*, and the longitudinal reinforcement ratio, ρ_l,s_, referring to the steel reinforcement. Depending on the load distance and the flexural reinforcement, the ranges of abscissa and ordinate were adjusted, which should be kept in mind while comparing the results. The strengthened specimens (solid curves) show less deflection than the plain RC specimens (dashed curves) at the same load level. The deflections, *w*, were measured continuously by means of displacement transducers beneath the specimens in the load axis.

For quantifying the strengthening effect, increases in ultimate loads were compared to the results of the corresponding non-strengthened reference tests. These quotients are indicated in Table 2. Particularly noteworthy are the shear tests S2-1, S7-1 and S7-2, which illustrate the considerable potential of SMART-DECK for increasing shear capacity. S2 and S7 only differ in the material combination of textile and mortar. The degree of strengthening of specimen S2 could even be increased with optimised materials in the reinforcing layer (specimen S7). The comparability of the results and the observed increases in shear capacity allow the conclusion that a simple top-side supplement of the flexural tensile reinforcement also has a positive influence on shear capacity.

In both partial tests on specimen S5 with a small steel reinforcement ratio, the type of failure shifted due to the strengthening with SMART-DECK. Capacity could be increased to such an extent that shear failure occurred instead of flexural failure.

**Figure 14:**
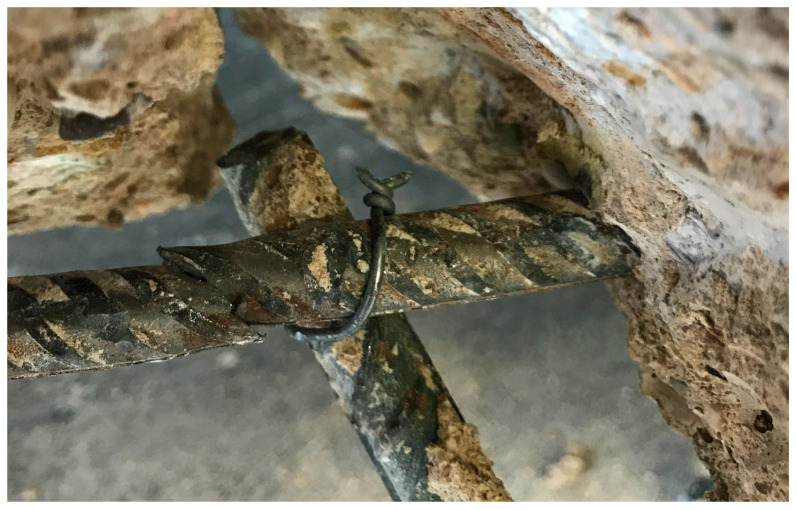
Plastic deformation of flexural tensile steel reinforcement in the cantilever section of S6 (photo by IMB, RWTH Aachen University).

While testing specimen S6, a loud popping noise occurred several times during the last quarter of the loading process, which was characterised by a slight load drop. This came along with the visible opening of a bending crack that appeared in the area of the cantilever support. Furthermore, the strain gauge attached to the flexural reinforcement in the RC base body failed after very high strains had been measured beforehand. The rebars in the support section (Figure 14) show that the steel reinforcement failed. Despite the partial opening of the interface, the stresses could clearly be redistributed that were released when the steel reinforcement reached its ultimate strength. The subsequent increases in loading provide the evidence that the released stresses could be taken completely by the TRC layer.

The results of the tests on S5 and S6 illustrate the enormous strengthening potential of SMART-DECK with regard to flexural capacity of bridge deck slabs in transverse direction. The strengthening degrees determined for the tests on S5 and S6 are beyond the values which could be achieved in bending tests within the previous experimental programme [78]. Since then, advanced materials for the strengthening layer could be provided by the project partners, resulting in better mechanical properties for the mortar and the textile. Delaminations in the concrete interface only occurred in case of pre-existing imperfections. Those represented the origin of cracks along the concrete interface at high-load levels. It was also noticed that such delaminations had a negative influence on the participation of the strengthening layer in shear load transfer (tests on S4), while high degrees of flexural strengthening remained possible (tests on S6). Therefore, it could be assumed that flexural capacity of the increased cross-section is relatively independent of the quality of the concrete interface. Fortunately, imperfections could be prevented later on by enhancing mortar and application method.

### 4.4. Results of Tests with Cyclic Loading

**Figure 15:**
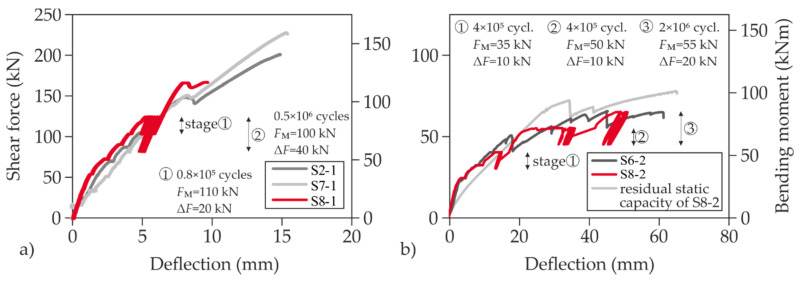
Comparison of load-deflection curves of cyclic (S8) and static tests (S2, S6 und S7). *F*_M_ = mean load, Δ*F* = peak-to-peak amplitude: shear test (**a**) and flexural test (**b**).

To investigate the influence of predominantly cyclic loads due to the impact of traffic, two tests were carried out under load collectives (red curves in Figure 15). At least 2 × 10^6^ load cycles were aimed at. S8 already showed imperfections of the interface in the test area of the first partial test prior to loading. Therefore, an initial load was applied that corresponded to approximately 75% of the failure load of the non-reinforced test. The first partial test featured a high longitudinal reinforcement ratio (shear test). Just as in the static tests, the load was applied stepwise. Subsequently, about 80,000 load cycles with an amplitude of 10 kN were applied at a frequency of *f* = 5.243 Hz. The upper load was 120 kN (maximum peak load) and the lower load 100 kN (minimum peak load). This load range corresponds to about 12.5% of the ultimate load of the RC reference specimen. Hardly any stress changes in the reinforcement were measured and there was no significant change in the crack pattern. Therefore, the amplitude was doubled while maintaining the upper load at 120 kN and approximately 0.5 × 10^6^ load cycles with a doubled load oscillation width were applied (lower load: 80 kN). In the meantime, the interface between the existing slab and the strengthening layer opened up, starting from the aforementioned imperfection that already existed before the start of the test. Nevertheless, no increase in the strain of the reinforcement and the concrete compression zone could be observed, which is why an increase in the average load was targeted.

**Figure 16:**
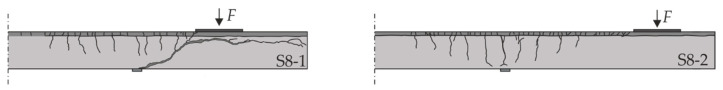
Crack patterns of cyclic tests on specimen S8.

Therefore, a lower load of 140 kN and an upper load of 160 kN were selected which was less and more than the capacity of the non-strengthened static reference test, respectively. However, shortly after reaching the upper load for the first time, a wide shear crack formed (Figure 16). It propagated horizontally at the level of the flexural steel reinforcement. The specimen thus failed due to interface failure and secondary flexural shear failure after only ≈0.6 × 10^6^ load cycles. Both the crack pattern and the ultimate load allow the conclusion that the interface damage prevented the strengthening layer to participate in the load transfer at high load level.

**Figure 17:**
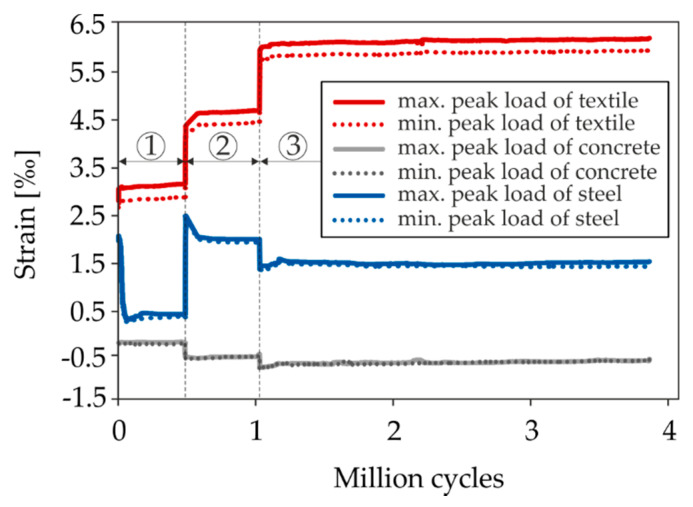
Development of the strains in the support axis during cyclic loading of test S8-2 referring to the peaks of the cycles.

The second cyclic partial test S8-2 was also performed as a load-collective test. In total, three load levels were applied (Figure 15b). First, the amplitude was retained at an average cylinder load of 35 kN and a load range of Δ*F* = 10 kN for approximately 4 × 10^5^ load cycles, whereby hardly any changes in stress occurred in the strengthening material. So, the mean stress was increased by 15 kN to 50 kN, which was already significantly higher than the capacity of the non-strengthened slab. During the following approximately 4 × 10^5^ load cycles at the second stage, no difference in the crack pattern and material stresses occurred (Figure 17), which is why the amplitude was doubled while the lower load remained the same.

The average load of the third cycle stage corresponded to approximately twice the capacity of the non-strengthened slab. At this stress level, the specimen was loaded up to a total number of more than 3 × 10^6^ load cycles. During the third cycle stage with more than 2 × 10^6^ load cycles, no significant increase in the material stresses could be determined, although clearly visible delaminations were found in the interface. Since the target number of load cycles was already exceeded, the load was taken off. Subsequently, the residual capacity was determined (Figure 15b).

Figure 17 shows a comparison of the strains measured during the test in the support axis (support at cantilever section) over the applied number of load cycles, whereby the three load levels are clearly visible. The strains of the steel reinforcement were determined using strain gauges (one measuring point averaged from two strain gauges), while the strains in the concrete compression zone and in the textile were determined using displacement transducers (approximation of the mean value from two measurements with a measuring length of 280 mm, >5 cracks in the strengthening layer, see above). The displacement transducers for determining the textile strain were located on the upper side of the slab segment, so the values shown are slightly higher than the actual strains in the textile plane.

**Figure 18:**
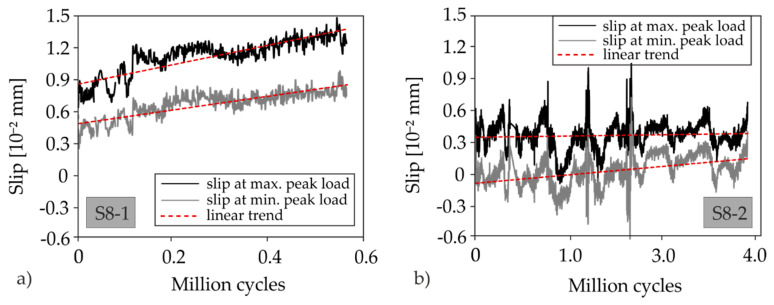
Measured development of the slip in the concrete interface compared to load duration of the cyclic tests: (**a**) shear test S8-1 and (**b**) flexural test S8-2.

In the course of each load level, an increase in concrete compression can be seen. A redistribution of the stresses from steel to textile can be assumed. During the shift from the first to the second load level, the static capacity of the non-strengthened slab was exceeded. The steel strain suggests that the rebars started yielding. During the following 10,000 load cycles, however, the strain decreases again. At the same time, an increase in strain in the textile occurs. When the stress is increased again to level 3, a decrease in stress is visible in the steel reinforcement, while the strain in the strengthening layer increases. It can be concluded that the entire additional stress due to the increase in the upper load is transferred by the textile. An examination of the cross-sections of both reinforcements shows that the equivalent textile area weighted to the tensile strength is more than eight times the steel cross-section. Despite the subsequently applied 2 × 10^6^ load cycles at high-load levels, no sign of fatigue failure of the steel reinforcement can be detected (e.g., disproportionate increase in strain). The specimen was prised open after the test to reveal the steel reinforcement in the support section of the cantilever. Slight confinement was visible which was much less distinct as in specimen S6 (Figure 14). This indicates that the carbon reinforcement indeed transferred the majority of the load during the last load stage. This suggests that it features good fatigue behaviour, which has been observed in other projects [40,79]. However, further investigations are required for verification. Furthermore, the presented tests could not provide any information on the fatigue behaviour of the interface. The separation of the strengthening layer from the RC base during loading was due to an already existing imperfection. Figure 18 shows the results of the slip measurements for the cyclic shear test S8-1 (Figure 18a) and the cyclic flexural test S8-2 (Figure 18b). The values refer to the head end of the slab segment and are the average values of two measurements with displacement transducers.

The slip during S8-1 increased continuously while the augmentation was less distinct in S8-1. Also, the total slip was larger in S8-1, which can be attributed to the higher load level of the shear test which lead to more pronounced stress in the interface. However, no conclusions can be drawn from those tests results regarding concrete-to-concrete bond with an intact interface. Considering it is an unreinforced interface, it is of particular importance to attest that an intact interface can be maintained despite fatigue loading.

## 5. Summary and Conclusions

This paper presented the results of tests on concrete bridge deck slabs with an additional layer of carbon textile-reinforced concrete. It is supposed to be applied between the existing structure and the road surface. This TRC layer (so called SMART-DECK) is intended to provide a monitoring system, preventive cathodic corrosion protection if necessary and the possibility to enhance the deck slab’s flexural and shear capacity in transverse direction of T-beam or hollow-core concrete bridges.

An experimental campaign was introduced comprising small- and large-scale tests. The small tests were conducted using TRC samples to investigate the interaction between suitable high-performance mortars and potential textile reinforcement materials aiming at material refinement and selection of proper materials and characterising its properties. The large-scale tests were conducted on slab specimens strengthened using SMART-DECK. They were partly statically or cyclically loaded until failure.

The outline of the specimens was varied by means of

- their longitudinal reinforcement ratio (steel flexural reinforcement on RC slabs representing the existing structure) and
- the bending moment-to-shear force ratio.

Therefore, load cases’ bending and shear could be addressed in twelve static and cyclic tests on strengthened specimens. A comparison to test results gained from non-strengthened reference specimens showed that SMART-DECK can enable high increases in capacity:

- An activation of the carbon concrete strengthening for existing slabs which feature shear failure allows strengthening degrees of 30–50%.
- In case of high bending loads on the existing slab, SMART-DECK can lead to an increase of the flexural capacity of 2.3 to 2.9 times the capacity of the non-strengthened RC member.
- In some cases, a shift from flexural to shear failure was observed by means of SMART-DECK. This corresponds to the maximum flexural strengthening of the referred RC component.

The results demonstrate the high potential of carbon concrete strengthening for ULS load cases in bridge deck slab design. Additionally, the fine crack pattern at the tensile side of the slab also resulted in significant advantages regarding serviceability.

For verification purposes, further investigations should be carried out, with the focus on fatigue loading with alternating loads and higher amplitudes typical for bridges. Further test results provide the basis for generalised design approaches which not only quantify the flexural but also the shear and fatigue strength.

## Figures and Tables

**Table 1:** Details of mortars and textiles.

Denotation	Specification
M-1-06	Base material with a maximum aggregate size of 6 mm
M-2-04	Base material with a maximum aggregate size of 4 mm
M-3-04	Advanced material based on M-2-04
M-4-04	Advanced material based on M-3-04
T-1-38	Base material with a mesh size of 38 mm
T-2-21	Base material with a mesh size of 21 mm
T-3-38	Advanced material based on T-1-38 with CNT *
T-4-38	Advanced material based on T-1-38 with modified epoxy resin

* CNT: Carbo Nano Tubes.

**Table 2:** Features of the large-scale tests.

Name	Target Failure Mode	Observed Failure	Material Combination Strengthening Layer	Load Distance	Longitudinal. Reinforcement Ratio	Concrete Compressive Strength ^##^	Mortar Strength	Strengthening Effect	Failure Load
				*a_i_* (m)	ρ_l,s_ (%)	*f*_cm,cyl_ (MPa)	*f*_cm,prism_/*f*_ct,fl_ (N/mm^2^)	η (-)	*F* (kN)
S1-1	V	V		0.7	1.0	41.0			155
S1-2	V	V		1.0	1.0	37.9			136
S1-2 *	V	V		1.0	1.0	41.0			145
S2-1	V	V	M3, T1	0.7	1.0	38.9	73.6/5.2	1.31	203
S2-2	V	I + V	M3, T1	1.0	1.0	38.9		1.03	144
S3-1	V	V		0.7	0.5	35.6			118
S3-2	V	V		1.0	0.5	35.6			110
S4-1	V	I + V	M4, T4	0.7	0.5	35.6	78.7/10.4	1.05	124
S4-2	V	I + V	M4, T4	1.0	0.5	35.6		1.08	119
S5-1	M	V	M4, T4	1.0	0.2	35.6		2.89	107
S5-2	M	V + I	M4, T4	1.3	0.2	35.6		3.63	103
S6-1	M	M + I	M4, T4	1.0	0.2	39.3		2.51	93
S6-2	M	M + I	M4, T4	1.3	0.2	39.3		2.30	65
S7-1	V	V	M4, T4	0.7	1.0	39.3		1.49	231
S7-2	V	V	M4, T4	1.0	1.0	39.3		1.53	215
S8-1 ^#^	V	I + V	M4, T4	0.7	1.0	39.3		1.03	160
S8-2 ^#^	M	M + I	M4, T4	1.3	0.2	39.3		2.11	78

^#^ Cyclic tests; ^##^ concrete strength of existing structure. * represents a repetition of S1-2.
